# Hysteroscopic Endometrial Ablation: From Indications to Instrumentation and Techniques—A Call to Action

**DOI:** 10.3390/diagnostics13030339

**Published:** 2023-01-17

**Authors:** Salvatore Giovanni Vitale, Luigi Della Corte, Michał Ciebiera, Josè Carugno, Gaetano Riemma, Ricardo Bassil Lasmar, Bernardo Portugal Lasmar, Ilker Kahramanoglu, Bulent Urman, Mislav Mikuš, Carlo De Angelis, Péter Török, Stefano Angioni

**Affiliations:** 1 Division of Gynecology and Obstetrics, Department of Surgical Sciences, University of Cagliari, 09124 Cagliari, Italy; 2 Department of Neuroscience, Reproductive Sciences and Dentistry, School of Medicine, University of Naples Federico II, Via Pansini 5, 80131 Naples, Italy; 3 Second Department of Obstetrics and Gynecology, Center of Postgraduate Medical Education, 00189 Warsaw, Poland; 4 Minimally Invasive Gynecology Unit, Obstetrics, Gynecology and Reproductive Sciences Department, Miller School of Medicine, University of Miami, Miami, FL 33136, USA; 5 Obstetrics and Gynecology Unit, Department of Woman, Child and General and Specialized Surgery, University of Campania “Luigi Vanvitelli”, 80128 Naples, Italy; 6 Department of Surgery and Specialities, Federal Fluminense University, Rio de Janeiro 24020-140, Brazil; 7 Department of Gynecologic Oncology, Emsey Hospital, 34912 Istanbul, Turkey; 8 Department of Obstetrics and Gynecology, Koc University School of Medicine, 34010 Istanbul, Turkey; 9 Department of Obstetrics and Gynecology, University Hospital Centre Zagreb, Petrova 13, 10000 Zagreb, Croatia; 10 Department of Maternal and Child Health and Urological Sciences, “Sapienza“ University of Rome, 00185 Rome, Italy; 11 Department of Obstetrics and Gynecology, Faculty of Medicine, University of Debrecen, H-4032 Debrecen, Hungary

**Keywords:** hysteroscopy, endometrium, ablation, endometrial ablation, hysterectomy, abnormal uterine bleeding, heavy menstrual bleeding, myoma, polyp, resectoscope

## Abstract

The development of minimally invasive techniques has led to the creation of innovative alternatives in cases where traditional methods are not applicable. In modern gynecology, hysteroscopy has become the gold standard for the evaluation and treatment of intrauterine pathology. Endometrial ablation (EA) is a procedure that uses different types of energy to destroy the endometrium and is currently used as an alternative technique in cases of heavy menstrual bleeding when medical treatment has failed and uterine preservation is desired. The aim of this review was to evaluate the feasibility, safety, and clinical outcomes of hysteroscopic EA as an alternative in patients with abnormal uterine bleeding. A detailed computerized search of the literature was performed in the main electronic databases (MEDLINE, EMBASE, Web of Science, PubMed, and Cochrane Library), from 1994 to June 2022, to evaluate the outcomes in patients with abnormal uterine bleeding (AUB) undergoing EA using hysteroscopic and non-hysteroscopic techniques. Only scientific publications in English were included. Twelve articles on the current use of endometrial ablation were included. Data on patient symptoms, tools used for EA, primary outcomes, and adverse events were recorded. EA should be considered an effective and safe approach in the management of patients with abnormal uterine bleeding caused by benign pathology, in whom medical treatment has failed or is contraindicated. Due to the lack of evidence, it would be interesting to determine whether EA would also have a role in the treatment of women with premalignant lesions, avoiding invasive surgical procedures or medical treatment in those patients for whom hysterectomy or the use of hormonal treatment is contraindicated.

## 1. Introduction

In recent decades, the development of minimally invasive techniques has provided therapeutic alternatives that are frequently used in clinical practice. In modern gynecology, hysteroscopy has become the gold standard for the evaluation and treatment of intracavitary lesions of the uterus [1,2,3,4,5,6,7,8]. Furthermore, the addition of new devices, such as the diode laser in hysteroscopy, has expanded the number of pathologies that can be treated [9,10,11,12,13,14]. Endoscopic instruments are used not only for the diagnosis [15], but also for the treatment of the patient with AUB. Currently, the incidence of preneoplastic lesions and endometrial cancer (EC) is increasing [16] and hysteroscopy with biopsy under direct visualization has demonstrated an elevated diagnostic accuracy (sensitivity: 96.55% and specificity: 100%) [17,18].

After the exclusion of premalignant or malignant conditions in women diagnosed with Abnormal Uterine Bleeding (AUB), endometrial ablation is considered a therapeutic option in patients who desire uterine preservation [19,20,21,22]. Endometrial ablation techniques destroy the entire endometrium down to the basalis membrane, causing a significant reduction of menstrual bleeding, frequently leading to amenorrhea [23]. However, concerns also have been raised when premalignant lesions are incidentally discovered after ablation [24]. 

AUB is one of the most common reasons for referral to gynecologic services, and leads to increased health care costs and decreased quality of life [23,24]. It affects up to 20% of women of reproductive age and is one of the most important symptoms of premalignant/malignant lesions in postmenopausal years [25,26]. For clinical purposes, it is defined as excessive menstrual blood loss that affects a woman’s physical, emotional, social, and material quality of life, and may occur alone or in combination with other symptoms. The International Federation of Gynecology and Obstetrics (FIGO) published the PALM-COEIN classification system in 2011, which classifies AUB according to etiology into structural and not structural entities [26]. The main objective of the treatment in patients with AUB is to decrease the amount of bleeding and associated morbidity, thus improving the quality of life. First-line management has traditionally consisted of medical treatments, such as NSAIDs, tranexamic acid, oral contraceptives, progestins, or progestin-releasing IUDs. The management of patients with AUB requires surgical treatment when medical options fail or in patients with contraindications to medical treatments. Hysterectomy is considered the “definitive” treatment for AUB, regardless of etiology, but has significant morbidity, a long recovery time, and high associated health care costs [27].

The aim of this review was to evaluate the feasibility, safety, and clinical outcomes of endometrial ablation as a therapeutic alternative in patients diagnosed with AUB. 

## 2. Materials and Methods

We adhered to the quality standards for narrative reviews, as defined and quantified by “SANRA—a scale for the quality assessment of narrative review articles” [28]. The relevant publications were identified after systematic queries of the following sources: PubMed, Google Scholar, Web of Science, and publishers’ databases, complemented by a cross-check of the reference lists. We used a combination of the search terms “endometrial ablation”, “endometrial resection”, “endometrial destruction”, with “hysteroscopy”, “resectoscope” and terms relevant to the topic of each paragraph (e.g., “indication”, “technique”, “instruments”). We did not apply any language restrictions.

**Figure 1:**
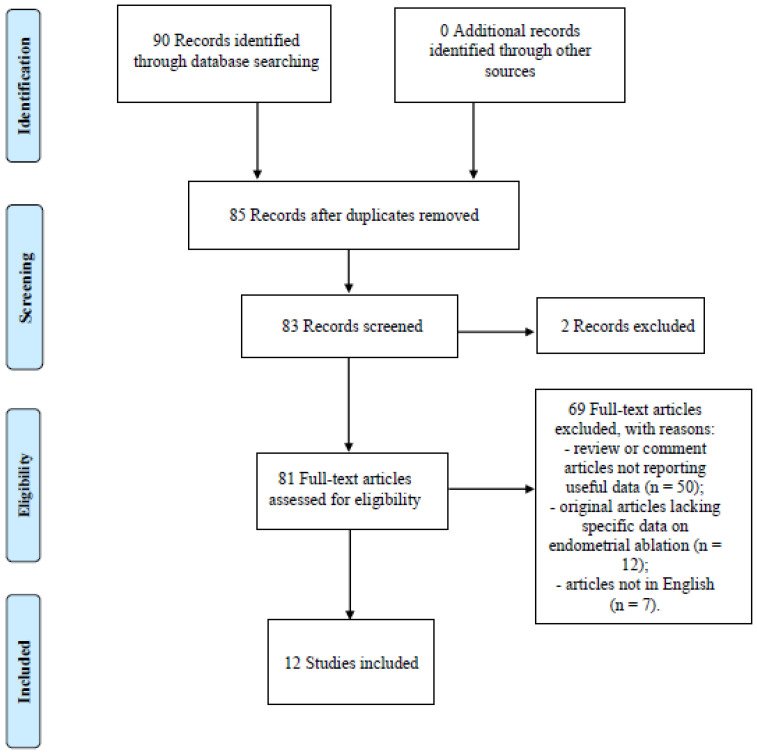
Flow diagram of narrative review search.

All articles describing the management of AUB applied to patients who had undergone endometrial ablation through hysteroscopic and non-hysteroscopic techniques were considered for review. Only original papers that reported specific experience data on the topic were included. Relevant aspects of every article were recorded and commented, with particular attention to the type of treatment applied and described outcomes. According to the etiologies classified in the PALM-COEIN system proposed by FIGO, the treatment for AUB includes hormones and other inflammatory mediators on the endometrium, in addition to antifibrinolytics. Frequently used therapies include: combined estrogen and progestogen; cyclic or continuous oral progestogen; injectable progestogen; levonorgestrel-releasing intrauterine devices (LNG-IUDs); non-steroidal anti-inflammatory drugs (NSAIDs); and tranexamic acid. Surgical treatment is indicated when medical therapy fails. The available surgical treatment options are endometrial ablation and hysterectomy. Outcomes analyzed included improvement in menstrual blood loss, impact on quality of life, duration of surgical procedure and length of hospital stay, time to return to work, adverse events, and requirements for repeat surgery due to failure of the initial surgical treatment. We included 12 articles in this review, while the remaining selected articles were used for a better understanding of the role of hysteroscopy in the treatment of patients with AUB (Figure 1).

## 3. Results

All studies stressed the importance of proper patient counseling before EA in particular, and considered the risks of recurrence or, in some cases, worsening of the symptoms such as pain and bleeding; as reported by Thomassee et al., hysterectomy after ablation was highest within the first 2 years after the initial procedure and it continued to increase up to 8 years post ablation, especially in those women younger than age 40 [29]. Most of the included papers were observational and/or retrospective studies based on routinely collected national data [30,31,32,33,34,35,36]. All studies showed a huge diversity regarding lifestyle and clinical factors, such as smoking status, body mass index, parity, type of delivery, size of the uterus, pre-existing genital prolapse, and previous urinary incontinence, as well as surgeon experience in EA [29,37,38,39]. Patient characteristics significantly associated with the development of post-ablation pain or bleeding were previous dysmenorrhea, prior tubal ligation, smoking, and an age less than 40 [30,33,34]. The role of endometriosis and adenomyosis is still uncertain [29]. A satisfaction greater than 65% regarding ablation techniques has been reported in most studies [32,34,37,39]. It is difficult to define the best tool to perform EA, but it seems to emerge that, when they are compared, the best results are obtained with bipolar energy: Herman et al. compared their randomized, controlled trial bipolar energy with thermal balloon reporting after 10-year follow-up rates of amenorrhea of 50/69 (73%) in the first group and 23/35 (66%) in the second group [RR. 1.1 (95% CI, 0.83–1.5)] [39].

In all of the included studies, adverse events were minimal or absent. Nevertheless, the results were highly heterogeneous and, in most cases, were not reported. Andersson et al. reported an allergic reaction during the procedure [38]. The best benefits are described in patients with higher anesthesiological risk who cannot carry out invasive surgical techniques [35]. In one case, the risk of endometrial and breast cancers and the hysterectomy rate after EA was evaluated: it was not associated with an elevated endometrial cancer [0.56 (95% CI 0.12–1.64)] or breast cancer [0.86 (95% CI 0.67–1.09)] risk and for breast cancer, while the presence of leiomyomas, young age, and history of prior cesarean deliveries or sterilization were associated with an increased risk of postablation hysterectomy in the Finnish patient cohort. Moreover, when EA was compared to hysterectomy, the advantages of the hysteroscopic approach were also associated with being less likely to undergo pelvic floor repair (adjusted HR 0.62; 95% CI, 0.50–0.77), insertion of tension-free vaginal tape (TVT) for stress urinary incontinence (adjusted HR, 0.55; 95% CI, 0.41–0.74), or genital fistula repair (adjusted HR, 0.18; 95% CI, 0.06–0.58) compared with the hysterectomy group [33]. Although in most cases these data were missing, the EA group had significantly shorter operation times [32,37,38] and lower complication rates [32,34,38,39,40]. All data in the included studies are reported in (Table 1).

## 4. Discussion

### 4.1. Clinical Indications of Endometrial Ablation: A Call to Action

Endometrial ablation is an effective treatment for heavy menstrual bleeding (HMB). Several devices for endometrial ablation have been developed worldwide, which are divided into two categories: hysteroscopic and non-hysteroscopic [41]. First-generation techniques, endometrial laser ablation (ELA), transcervical resection of the endometrium (TCRE), which uses the resectoscope, and rollerball endometrial ablation (RBEA), require direct visualization of the uterine cavity with a hysteroscope during the procedure, as opposed to second generation non-hysteroscopic techniques, that blindly destroy the endometrium by various methods, including thermal balloon endometrial ablation (TBEA), microwave endometrial ablation (MEA), hydrothermal ablation (HTA), bipolar radiofrequency endometrial ablation, and endometrial cryotherapy. The nature of some of the abovementioned devices allows patients to be treated under local anesthesia, which makes them cost-effective [42,43,44]. 

Although continued bleeding is the most common sign of ablation failure, postablation pain has been described after ablation procedures [45]. Thomassee et al. found no significant differences in clinic characteristics in patients who experienced pain after endometrial ablation. Among different types of ablation techniques, the risk of developing pain after thermal balloons is 25.4%, and after bipolar radiofrequency ablation methods the risk is 16.0% (adjusted OR 1.27, 95% CI 0.59–1.69; *p* = 0.99) [29]. 

The goal of endometrial ablation is to achieve reduction of menstrual bleeding due to destruction of the endometrium. This technique significantly reduces menstrual blood loss to a level that is acceptable for most patients [26]. They are frequently combined with endometrial suppressive drugs used before ablation to maximize depth of destruction. Indeed, the use of endometrial suppressive drugs (danazol and gonadotropin-releasing hormone analogs) before ablation favors an adequate depth of destruction and is recommended to increase the postoperative amenorrhea rate [44]. 

Hysteroscopic endometrial ablation may be an effective alternative to resection for the treatment of AUB. Helleland et al. reported a comparable satisfaction rate with the procedure in patients undergoing endometrial ablation and those undergoing hysteroscopic endometrial resection, with a shorter procedure time, a median of 13 min (95% Confidence Interval (CI) 12–14)) compared to a median of 25 min (95% CI 23–26), and a lower risk of complications: around 2% vs. 13% as in the case of resection [32]. As mentioned above, AUB is a common gynecological complaint affecting up to one-third of women of reproductive age. Overall, AUB not responding to medical treatment represents 23% of the indications for hysterectomy [26,27]. Moreover, preoperative anemia is an independent risk factor for adverse outcomes after surgery [45,46,47,48,49,50]. Taking into consideration the abovementioned problems, minimally invasive techniques have been introduced as they are associated with fewer complications, require shorter recovery time, and are better tolerated by the patients [33]. 

Although medical treatments are preferred as first line management options [30,51,52], a recent Cochrane review showed that over five years, 77% of patients who were medically treated for AUB subsequently underwent surgical treatment [53]. Thus, medical treatment is burdened with a high failure rate. Hysterectomy remains the definitive surgical treatment for AUB; however, it is associated with a higher risk of pelvic floor organ prolapse and the need for pelvic floor repair [adjusted hazards ratio, 0.62; 95% CI: 0.50, 0.77] and surgery for stress urinary incontinence (adjusted hazards ratio, 0.55; 95% CI, 0.41, 0.74), when compared with endometrial ablation [34]. Nevertheless, Pinion et al. found a significantly higher satisfaction rate in women with dysfunctional uterine bleeding treated with hysterectomy (89%) compared to those who were treated with hysteroscopy (78%) (*p* < 0.05). Hysteroscopy was associated, however, with a lower morbidity rate and a significantly shorter recovery period [34]. Moreover, serious complications associated with hysterectomy, such as bowel injury, urinary tract injury, and cardiovascular and respiratory complications, are quite rare [39,49,50].

Hysteroscopy is frequently performed in the outpatient setting [51]. Office hysteroscopy is a highly effective diagnostic and therapeutic procedure, especially in women of reproductive age [4]. Outpatient endometrial ablation without the need of general anesthesia can also be performed with low failure rates [52,53]. In order to assess if office hysteroscopy is a well-tolerated procedure, different studies have been performed to evaluate the feasibility and safety of hysteroscopy in the outpatient setting [54,55]. 

Currently, office hysteroscopy is considered the gold standard procedure for the treatment of intrauterine pathology. In-office hysteroscopy is associated with a lower risk of complications, such as perforation, in comparison with when it is performed in the operating room under general anesthesia, with a rapid recovery time and early return to activities [3].

### 4.2. Tools and Techniques

The resectoscope is widely used for hysteroscopic endometrial resectoscopic ablation. It employs a 26–27 Fr gauge (8.7–9 mm) working element, a thin telescope of 4 mm, and it is also equipped with an electrical wire loop, rollerball, or spiked-ball tip. The instrument is inserted into the uterine cavity after cervical dilatation up to 9 mm [56]. Smaller resectoscopes are available (22 Fr gauge, 7.3 mm), also requiring cervical dilatation prior to introduction into the uterine cavity. The cavity is distended with a nonconductive hypo-osmolar or physiological solutions, such as normal saline, depending on the type of energy used, and the fluid is instilled under manometric control, with a pressure generated by a pneumatic cuff and a vacuum applied for suction. The use of normal saline as distention media is associated with less postoperative pain when compared to CO^2^ [57], and it also allows the use of bipolar energy. After careful inspection of the cavity, the endometrium is resected with a cutting loop with monopolar or bipolar energy. This technique is usually performed in an operating room under general anesthesia. The risk of complications is increased by the need to perform cervical dilatation, which may also lengthen the operating time.

Conversely, the mini-resectoscope has an outer diameter of 5 mm with a 2.9 mm telescope that does not require cervical dilatation to insert it into the uterus. As a result of this, the use of mini-resectoscopes is associated with lower complications rates, as well as less intraoperative and postoperative morbidity [58]. In this regard, Papalampros et al. conducted a prospective observational study to evaluate the feasibility and the safety of a 16 Fr mini-resectoscope for the surgical treatment of endometrial polyps and small submucous fibroids in the outpatient setting. No intra or postoperative complications were reported, and all procedures were successfully conducted. In all of the patients included in the study, the lesions were completely removed without the need for general anesthesia [59]. A pilot study that compared the mini-resectoscope to the conventional resectoscope for hysteroscopic adhesiolysis in infertile women showed that the use of the mini-resectoscope was associated with less postoperative pain and a shorter operating time, due to the lack of the need for cervical dilation [60]. In terms of prevention of intrauterine adhesion formation, hyaluronic acid seems to be of great importance, but the lack of a clear best therapy suggests the need for further studies [61]. In addition to the mini-resectoscope, the mini-hysteroscopy, characterized by a 2.7-mm outer diameter telescope with a 3.5-mm outer diameter single-flow diagnostic sheath, can be very useful in cases of outpatient diagnostic hysteroscopy. Indeed, Cicinelli et al. demonstrated how the rate of successful insertion into the uterine cavity and optimal endometrial evaluation (99.52% vs. 72.53% and 98.53% vs. 92.33%, respectively) as well as pain and vaso-vagal reaction (0.10 ± 0.34 vs.1.09 ± 0.53 and 2.25% vs. 17.12%) were significantly lower in the mini-hysteroscopy group compared to the traditional hysteroscope, highlighting that mini-hysteroscopy with a vaginoscopic approach is a very well-tolerated, effective, and safe outpatient procedure [62].

Technological advances and improvements in surgical techniques have expanded the indications of office hysteroscopy; however, it is still dependent upon user experience and the extent of intrauterine pathology [63]. Small submucous myomas can be removed in the office setting, but may require more than one procedure [64,65,66,67].

A fairly recent device used for performing hysteroscopic surgery is based on mechanical tissue removal [68,69,70,71,72]. It crushes the intracavitary lesions into small pieces with a mechanical rotating blade and simultaneously evacuates these pieces from the uterine cavity with a built-in suction [15]. 

A retrospective study comparing hysteroscopic tissue removal systems to the conventional resectoscope for the removal of intrauterine myomas and polyps showed the mean operating time to be shorter when using tissue retrieval systems. There was no difference in the rate of complications [7,22,73,74]. 

All of the abovementioned devices are used for the treatment of intrauterine lesions, such as polyps, myomas, and other endometrial anomalies. The instrument selection and technique used to treat intrauterine pathology is based on personal experience or the surgeon’s preferences. Here, it should also be emphasized that in many cases it is the availability of the adequate tool that determines the success of the procedure, as is the case, for example, with a biopsy device [69]; therefore, one should always seek the optimal tool to perform the requested task [70].

All of the abovementioned hysteroscopic tools can destroy the endometrium and subsequently perform a complete endometrial ablation. In addition, several devices have been conceptualized to perform a non-resectoscopic endometrial ablation (NREA). Currently, several NREA devices that use a variety of energy sources are approved for clinical use, including hot fluid freely circulating in the uterine cavity, bipolar radiofrequency ablation, cryotherapy, and fluid encapsulated in a balloon or Argon gas to produce plasma energy and radiofrequency energy [70].

When analyzing the results of surgeries using non-resectoscopic devices, the patient satisfaction rate is high and re-intervention rates are low, even when amenorrhea is not achieved. As evidenced by the FDA’s pivotal trials, which revealed satisfaction ratings of 86% to 99% at 1 year, all of these devices appear to be highly effective and produce high levels of patient satisfaction [70]. 

There are few direct comparisons of non-resectoscopic devices, and it is difficult to evaluate outcomes between trials due to variations in outcome measurements, preoperative endometrial preparation protocols, nine practice environments, and follow-up timeframes. Radiofrequency and fluid-encapsulating devices have received the greatest research attention in randomized studies [70].

### 4.3. Current Indication for Complete Endometrial Ablation 

Currently, endometrial ablation is indicated in women of childbearing age with heavy menstrual bleeding of benign etiology [26]. This minimally invasive approach has gained approval among physicians as NICE recommends it as an alternative for women with AUB without uterine abnormalities or fibroids <than 3 cm in diameter [48]. Moreover, others have suggested that endometrial ablation is preferable to hysterectomy for women with AUB when the endometrial cavity is <10 cm in length [41]. After treatment, amenorrhea is achieved in 14–70% of cases. Furthermore, surveys conducted after surgery showed how both quality of life and symptoms improved within 12 months after treatment [71].

For women with increased surgical risks or with contraindications for medical treatment due to pre-existing comorbidities, endometrial ablation may be a minimally invasive therapeutic option for the treatment of heavy menstrual bleeding [72]. Although endometrial ablation is a minimally invasive procedure, it is not free from complications. In this regard, the Medicines and Healthcare products Regulatory Agency (MHRA) recently published guidelines on the responsibilities of manufacturers, regulators, and clinicians regarding the safety of endometrial ablation devices [73]. The reported rate of complications with hysteroscopic surgery was relatively low, about 0.28%. Overall, complications with hysteroscopic surgery include uterine perforation, fluid overload, gas embolism, thermal injury, excessive bleeding, intrauterine adhesions formation, and infection. The most common complications, bleeding and uterine perforation, are easily managed without complex intervention [49,73,74]. The need for additional surgery due to perioperative complications is rare [23]. Furthermore, approximately half of the complications were at the time of introducing the hysteroscope, which is minimized if cervical dilation is not needed [75]. Di Spiezio Sardo et al. investigated the success of outpatient hysteroscopy in 5000 women, evaluating the correlation between the type of approach (traditional vs. vaginoscopic) and the type of caliber hysteroscopes (3.5 mm vs. 5 mm), which suggested that the success of outpatient hysteroscopy was positively affected by the use of smaller caliber hysteroscopes, as well as using the vaginoscopic approach. The need for cervical dilatation was associated with an increased failure rate [50]. Patients at an age less than 45 years, having had more than five pregnancies, a history of tubal sterilization, obesity, and a history of dysmenorrhea were predictors of endometrial ablation failure [37,74].

A prospective observational study evaluated the use and efficacy of thermal balloon ablation (TBA) of the endometrium in the outpatient setting in 53 women with heavy menstrual bleeding not responding to medical treatment. All procedures were performed with local anesthesia. The procedure was successfully completed in 50 of 53 women (94% of cases). A reduction in menstrual flow was experienced by 80% of the subjects, and a positive satisfaction rate was reported by 67% [37]. A subsequently performed similar study reported on 56 women who underwent TBA in the outpatient setting. The procedure was completed in 97% of women (54 out of 56 patients). Only 3 out of 54 patients had short-term complications, but there were no perforations or other serious complications. Indeed, improvement in menstrual bleeding was reported by 34 (81%) women. Accordingly, TBA seems to be safe and effective for the treatment of HMB, as well as being safe to be performed in an outpatient setting [38]. When compared, after 10 years of follow-up, TBA and bipolar endometrial ablation in premenopausal women suffering from heavy menorrhagia using the bipolar technique were not superior to balloon ablation; interestingly, unlike what was reported at 1 and 5 years of follow-up [39]. Furthermore, Abbott et al., in a descriptive cohort study, evaluated 139 women within one year of treatment who had undergone endometrial ablation by one of following methods: TBA (Cavaterm), endometrial laser interstitial thermotherapy (ELITT), endometrial laser ablation (ELA), or NovaSure impedance-controlled system [71]. No significant differences, in terms of effectiveness, patient satisfaction, and patient safety, with either first- or second-generation endometrial ablation devices were found, but newer techniques were associated with shorter operating times compared to the first generation [71]. However, the quality of life of women with AUB was improved after treatment, regardless of the ablation modality that was used [41,71]. Endometrial ablation is also feasible in women with AUB due to ovulatory dysfunction as an alternative to hysterectomy. The treatment failure rate was 11.7% at 5 years (95% confidence interval [CI], 6.5–16.9%) [40].

As more procedures have been performed in recent decades, incidental findings of premalignant lesions after procedures have been reported in the literature [12,24,76]. This fact raises concerns. Malignant lesions are a contraindication to the procedure. Recent findings seem to demonstrate a similar risk of endometrial cancer between women treated with endometrial ablation and the normal population. Therefore, the management of these women could be a thought-provoking issue that opens up new research scenarios.

### 4.4. Limitations and Contraindications 

Not all patients are eligible for endometrial ablation. It should not be performed during menopause, and it is not recommended for women with a desire for future fertility, a history of endometrial hyperplasia or cancer, or confirmed cervical cancer. Exclusion criteria vary for every specific ablative device; previous uterine surgery, such as classical cesarean section or transmural myomectomy, as well as an irregular cavity with congenital defects, submucous myomas with significant cavity distortion, a uterine cavity with a diameter greater than 11 cm, are all relative contraindications for endometrial ablation [23,48].

This study has the great strength to represent a wide overview of indications, instrumentation, and techniques of hysteroscopic endometrial ablation that could provide an useful guide for physicians who want to approach a technique that, although niche, has seen widespread use in recent years. On the other hand, it has some limitations: its narrative nature precludes a quantitative analysis through a systematic analysis because of the heterogeneity of the data published so far, which makes a clear assessment of the best tool to perform EA difficult, as well as to assess the adverse events after the procedure. 

### 4.5. Future Perspectives: Endometrial Ablation for Preneoplastic Lesions 

Overall, the role of hysteroscopy in the diagnosis of uterine cancer is well accepted [77,78,79,80]. Hysteroscopy is a more precise technique for the diagnosis of endometrial cancer, with a sensitivity and specificity of 96% and 100%, respectively [1]. Nevertheless, hysteroscopic ablation is still not universally considered acceptable for the treatment of early malignant endometrial carcinoma. Atypical endometrial hyperplasia (AEH) or EIN may occur after endometrial ablation, and the burden of potential treatment has been documented. Cooper et al. analyzed the rate of gynecologic cancer during the follow-up of 37,120 women who underwent hysterectomy and 11,299 women treated with endometrial ablation due to AUB. Cancer occurred in less than 2% of the patients [584 (1.57%) and 130 (1.15%) patients who underwent hysterectomy and endometrial ablation, respectively; adjusted hazards ratio, 1.14; 95% CI, 0.93–1.39]; the most frequent type was breast cancer [33]. This is noteworthy because it contrasts with the use of a levonorgestrel-releasing intrauterine system, which has been documented to increase the risk of breast cancer [1,31,81]. Since the risk of endometrial cancer is similar to that in the general population and that endometrial hyperplasia has been previously reported as an indication for endometrial ablation [81], the range of indications for this minimally invasive approach could also be expanded in special circumstances. It may be interesting to investigate whether endometrial ablation could be useful in the treatment of premalignant lesions to preserve the uterus and avoid hysterectomy in patients for whom surgery or the use of hormonal treatment is a contraindication [82].

## 5. Conclusions

The role of hysteroscopy in the diagnosis of endometrial diseases and, in particular, endometrial cancer is clear. Available data suggest that hysteroscopy is a reliable technique for its diagnosis. Nevertheless, surgical hysteroscopy (resectoscopic ablation) is still not widely accepted for the treatment of early malignant endometrial cancer. Further studies are needed to determine if resectoscopic endometrial ablation is a safe alternative for the treatment of premalignant lesions or early-stage endometrial cancer.

We believe that hysteroscopic resectoscopic endometrial ablation could be considered an effective and safe approach in the management of patients with AUB when more invasive surgical options are to be avoided and medical treatment is contraindicated or has previously failed. Furthermore, the different techniques that characterize the surgical management of AUB should be further compared to identify the optimal treatment and to improve satisfaction rates and outcomes. Another important aspect to consider is the length of follow-up, which has varied in the studies published so far. However, long-term outcomes, including the re-intervention rate and the improvement in menstrual blood loss, need to be monitored. 

Further studies are needed to collect reliable data and evaluate the feasibility and safety of endometrial ablation as an acceptable alternative for patients with premalignant endometrial lesions and contraindications to hysterectomy or medical therapy.

## Figures and Tables

**Table 1:** Characteristics of the included studies.

Study	Country and Year	Design	Population	Symptoms	Tools Used for EA *	Primary Outcome	Menstrual Blood Loss	Impact on Quality of Life	Duration of Surgical Procedure	Length of Hospital Stay	Time to Return to Work	Adverse Events	Requirements for Repeat Surgery °^§^
Soini [30]	Finland 1997–2014	Retrospective cohort study	5484 (*n*)	AUB Dysmenorrhea	NR	Risk of endometrial and breast cancer and the hysterectomy rate *after* EA	4488 (*n*) NR *after* EA	NR	NA	NA	NA	NA	6 (*n*) EA 1086 (*n*) Hysterectomy
Wishall [31]	United States of America 2003–2016	Retrospective cohort study	300 (*n*)	AUB Dysmenorrhea	Thermal balloon Radiofrequency bipolar Microwave Hydrothermal	Risk factors for postablation pain or hysterectomy	NR 23 (*n*) *after* EA	NR	NA	NA	NA	NA	51 (*n*) Hysterectomy
Thomassee [29]	United States of America 2006–2010	Retrospective cohort study	437 (*n*)	AUB Dysmenorrhea	Rollerball Thermal balloon Radiofrequency bipolar	Postoperative pelvic pain	NR 66 (*n*) *after* EA	NR	NA	NA	NA	NA	0.2 (%) EA 65 (*n*) Hysterectomy
Helleland [32]	Norway 1992–2014	Retrospective cohort study	135 (*n*)	AUB Dysmenorrhea	Radiofrequency bipolar	Efficacy of EA	20 (*n*) 5 (*n*) *after* EA	Satisfaction rates 73–85 (%)	13 min	NA	NA	1 (technical problems)	11 (*n*) Hysterectomy
Cooper [33]	United Kingdom 1989–2006	Retrospective cohort study	14,078 (*n)*	AUB Dysmenorrhea	NR	Risk of further gynaecological surgery and cancer	14,078 (*n)* 2779 (*n*) *after* EA	NR	NA	NA	NA	NA	379 (*n*) EA 2779 (*n*) Hysterectomy
Pinion [34]	United Kingdom 1990–1992	Retrospective cohort study	53 (*n*)	AUB Dysmenorrhea	Laser ablation	Efficacy of EA and Satisfaction	26 (*n*) 5 (*n*) *after* EA	Satisfaction rates 70–90 (%)	44 min	2–5	NA	1 (bowel damage)	11 (*n*) EA 17 (*n*) Hysterectomy
Ajao [35]	United States of America 2006–2014	Retrospective cohort study	634 (*n*)	AUB Dysmenorrhea *r*	Radiofrequency bipolar Thermal balloon	Safety in patients with high-risk ASA	NR NR *after* EA	NA	NR	NA	NA	NR	81 (*n*) Hysterectomy
Smithling [36]	United States of America 2007–2009	Retrospective cohort study	968 (*n*)	AUB Dysmenorrhea	Radiofrequency bipolar	Risks of treatment failure	931 (*n*) 40 (*n*) *after* EA	NA	NA	NA	NA	NA	11 (*n*) EA 74 (*n*) Hysterectomy
Clark [37]	United Kingdom 2001–2003	Prospective observational study	53 (*n*)	AUB Dysmenorrhea	Thermal balloon	Efficacy of EA	39 (*n*) 6 (*n*) *after* EA	Satisfaction rates 67 (%)	8 min	NA	NA	1 (*balloon blockage*)	1 (*n)* EA 3 (*n*) Hysterectomy
Andersson [38]	Sweden 2001–2005	Prospective observational study	54 (*n*)	AUB Dysmenorrhea	Thermal balloon	Efficacy of EA	42 (*n*) 8 (*n*) *after* EA	NA	15 min	NA	NA	3 (*allergic reaction, balloon blockage, abdominal cramps*)	1 (*n*) Hysterectomy 3 (*n*) Hysteroscopical endometrial resection
Herman [39]	Netherlands 1999–2011	Double-blind RCT	126 (*n*)	AUB Dysmenorrhea	Thermal balloon Radiofrequency bipolar	Thermal balloon vs. Bipolar	126 (*n*) 53 (*n*) *after* EA	Satisfaction rates 77–81(%)	NA	NA	NA	NA	21 (*n*) EA 2 (*n*) Hysterectomy
Hokenstad [40]	United States of America 1998–2005	Prospective observational study	711 (*n*)	AUB Dysmenorrhea	Thermal balloon Radiofrequency bipolar	Efficacy of EA	489 (*n*) 66 (*n*) *after* EA	NA	NA	NA	NA	NA	66 (*n*) Hysterectomy

* EA: endometrial ablation; °^§^ due to failure of the initial surgical treatment; AUB: abnormal uterine bleeding; RCT: randomised controlled trial; NR: not reported; NA: not applicable.

## Data Availability

Not applicable.
